# The Formation of Two Hybrid Plasmids Mediated by IS*26* and Tn*6952* in *Salmonella enterica* Serotype *Enteritidis*

**DOI:** 10.3389/fmicb.2021.676574

**Published:** 2021-05-28

**Authors:** Ying-ying Liu, Dan-dan He, Meng-ke Zhang, Yu-shan Pan, Hua Wu, Li Yuan, Jian-hua Liu, Gong-zheng Hu

**Affiliations:** College of Animal Husbandry and Veterinary Science, Henan Agricultural University, Zhengzhou, China

**Keywords:** *Salmonella enterica*, cointegrate plasmid, IS*26*, Tn*6952*, IncN1-F33:A–:B– plasmid

## Abstract

To characterize the formation mechanism and characteristics of two cointegrate plasmids in *Salmonella enterica* serotype *Enteritidis* strain S13, plasmids from strain S13 and three corresponding transconjugants were subjected to whole genome sequencing and analyzed using bioinformatics tools. The traits of two fusion plasmids in transconjugants were characterized by stability and conjugation experiments. Sequence analysis indicated that strain S13 contained four plasmids, including *mcr-1*-bearing pS13-1, *bla*_CTX–M–55_-carrying pS13-2, *tet*(M)-bearing pS13-3, and *floR*-carrying pS13-4. IncN1-F33:A–:B– plasmid pS13-2, respectively, fused with IncFI:A–:B– plasmid pS13-3 and IncX1 plasmid pS13-4, which generated two cointegrate plasmids, designated pS13D and pS13F, which involved in two intermolecular replicative mechanisms mediated by IS*26* and the novel transposon Tn*6952* (*Δ*Tn*AS3*-IS*26*-*Δ*IS*Ecp1*-*ramA*-*Δ*IS*26*-*Δ*Tn*AS1*), respectively. This is the first report of the fusion of the IncN1-F33:A–:B– plasmid and IncFI:A–:B– plasmid mediated by IS*26*, and with IncX1 plasmid mediated by Tn*6952.* The formation and evolution of cointegrate plasmids could expand the resistance and host spectrum of fusion plasmids.

## Introduction

*Salmonella* is an important zoonotic intestinal pathogen and a leading cause of microbial food poisoning (Scallan et al., 2011). In recent years, the widespread application of antibiotics in clinical practice is the driving force of the resistance of *salmonella* to antimicrobial agents (Deng et al., 2012). The acquisition of genetic material, such as integron gene cassettes, transposons, and resistance plasmids, is the main reason for the rapid development of multidrug resistance (MDR) in *Salmonella* (Hsu et al., 2006; Wright, 2007; Hu and Li, 2009).

The emergence and spread of fusion plasmids in *Enterobacteriaceae* pose great public concerns. Conjugative plasmids can capture MDR non-conjugative plasmids through replicative transposition of insertion sequences, such as IS*1*, IS*26*, and IS*kpn19*, thereby expanding the host range of the plasmids (Ohtsubo et al., 1981; He et al., 2015; Chen et al., 2017; Xie et al., 2018; Li et al., 2020). In the present study, the conjugative CTX-M-producing IncN1-F33:A–:B– plasmid was captured by two non-conjugative plasmids in *Salmonella*, resulting in the rapid transmission of the antibiotic resistance genes *tet*(M) and *floR*. F33:A–:B– plasmids are epidemic in *Escherichia coli* and widely disseminated by acquiring other resistance genes, replicon genes, and plasmids (He et al., 2019). Here, two hybrid plasmids mediated by IS*26* and Tn*6952* were first characterized in the same *Salmonella enterica* serotype *Enteritidis*. Moreover, the traits of two fusion plasmids in transconjugants were further investigated by stability and conjugation experiments.

## Materials and Methods

### Bacterial Strains

In 2016, a clinical *tet*(M)-positive *Salmonella enterica* serotype *Enteritidis* strain S13 was isolated from a pig in a livestock premises during surveillance of the *tet*(M) gene in the Henan Province, China. The strain was identified by the VITEK 2 automated identification system (bioMérieux, Marcy-l’Étoile, France) and serotyped according to the Kauffmann–White scheme. Multilocus sequence typing (MLST) of strain S13 was identified according to the protocol recommended at http://mlst.warwick.ac.uk. The genes *tet*(M), *mcr-1*, *bla*_CTX–M–55_, and *floR*, and the genetic environment of *tet*(M) in strain S13 were characterized by polymerase chain reaction (PCR) analysis with the use of the primers listed in Supplementary Table 1.

### Antimicrobial Susceptibility Testing

Antimicrobial susceptibility testing to 12 antibiotics of *Salmonella* strain S13 and its transconjugants was performed using the broth microdilution method and interpreted in accordance with the Clinical and Laboratory Standards Institute, (2017) guidelines. For florfenicol, the resistant breakpoint was interpreted in accordance with the guidelines proposed by the European Committee on Antimicrobial Susceptibility Testing^1^. *E. coli* strain ATCC 25922 was used for quality control.

### Conjugation Experiments, S1 Nuclease-Pulsed Field Gel Electrophoresis (S1-PFGE), and Southern Blot Hybridization

Conjugation experiments were performed utilizing rifampicin-resistant *E. coli* strain C600 as the recipient in order to assess the transferability of plasmids to *S*. *enterica* strain S13. Four different transconjugants were selected on MacConkey agar plates supplemented with rifampin (400 mg/L) and doxycycline (16 mg/L) or florfenicol (16 mg/L), ceftiofur (16 mg/L), and colistin (2 mg/L). The plasmid profiles of the donor and transconjugant strains, and the location of the *tet*(M) gene were determined by S1-PFGE and southern blot hybridization. The transfer frequency was calculated as the ratio of the number of transconjugants per recipient. All transconjugants were confirmed by PCR analysis as described previously (Sun et al., 2019).

### Whole Genome Sequencing (WGS) and Plasmid Analysis

To investigate alterations to the sizes of the plasmids and their genetic contexts in the donor and transconjugant strains, whole plasmid DNA of strain S13 and the corresponding transconjugants S13D, S13F, and S13S were extracted using the Qiagen Plasmid Midi Kit (Qiagen, Hilden, Germany) and sequenced with the NextSeq 500 Sequencing System (Illumina, Inc., San Diego, CA, United States) and the MinION nanopore sequencing device (Oxford Nanopore Technologies, Oxford, United Kingdom). The complete genome and plasmid sequences were assembled with the Unicycler 0.4.4 assembly pipeline (Wick et al., 2017; Li et al., 2018). The NCBI Prokaryotic Genome Annotation Pipeline (PGAP) and the RAST tool were used to annotate the completed plasmid sequence (Aziz et al., 2008). The plasmid sequences were compared with BRIG and Easyfig tool (Alikhan et al., 2011; Beatson, 2011).

### Cointegration Assay and Plasmid Stability

To assess the self-transferability of two fusion plasmids, pS13D and pS13F, two cointegration assays were performed *via* conjugation utilizing the C600 transconjugants S13D and S13F as the donor, respectively, and azide-resistant *E. coli* J53 as the recipient. The conjugation frequency was calculated as the number of transconjugants per recipient. The stability of fusion plasmids pS13D and pS13F were assessed as described previously (Liu Y. Y. et al., 2020).

### Nucleotide Sequence Accession Number

The complete sequences of plasmids pS13-1, pS13-2, pS13-3 (pS13S), pS13-4, and chromosome in S13 and the fusion plasmids pS13D and pS13F were submitted to the GenBank under accession numbers CP047090, CP047091, CP047092, CP047093, CP047094, MT657397, and MT742153, respectively.

## Results and Disscussion

### Characterization of *tet*(M)-Bearing *S. enterica* Stain S13

The *S. enterica* stain S13 carrying *bla*_CTX–M–55_, *tet*(M), *floR*, and *mcr-1* genes was resistant to amoxicillin, ceftiofur, cefquinome, tetracycline, oxytetracycline, florfenicol, colistin, and sulfamethoxazole-trimethoprim (Supplementary Table 2). S1-PFGE showed that S13 harbored four plasmids, designated as pS13-1 (~96 kb), pS13-2 (~89 kb), pS13-3 (~77 kb), and pS13-4 (~30 kb) (Supplementary Figure 1). Conjugation experiments revealed that the *tet*(M)-bearing transconjugant S13D harboring a single plasmid with ~167 kb in size, pS13D, the *floR*-bearing transconjugant S13F carrying the ~128-kb plasmid pS13F, and the *bla*_CTX–M–55_-bearing transconjugant S13S bearing the ~89-kb plasmid pS13S had transfer frequencies of 5.36×10^–5^, 2.11×10^–6^, and 1.06×10^–4^, respectively, while no *mcr-1*-carrying transconjugant was obtained despite repeated attempts (Table 1). The *tet*(M) gene was located on plasmids pS13-3 and pS13D (Supplementary Figure 1).

**TABLE 1:** The characterizations of plasmid carried by strain S13 and its transconjugants.

Strain	Name	No. plasmids (name)	Size (kb)	Source	Conjugation frequencies	Replication type	Resistance genes
Parent strain	S13	4, (pS13-1, pS13-2, pS13-3, pS13-4)	~96 ~89 ~77 ~30	–	–	– IncN1-F33:A–:B– IncFI:A–:B– IncX1	*mcr-1 bla*_CTX–M–55_*tet*(M), *oqxAB, aadA1, aadA2, bla*_*TEM*_ *floR*
Transconjugants	S13D	1, (pS13D)	~167	pS13-2 pS13-3	5.36×10^–5^	IncN1–F33:A–:B– IncFI:A–:B–	*tet*(M), *oqxAB, aadA1,aaDA2*, *bla*_*TEM*_ and *bla*_CTX–M–55_
	S13F	1, (pS13F)	~128	pS13-2 pS13-4	2.11×10^–6^	IncN1-F33:A–:B– IncX1	*floR*, *bla*_CTX–M–55_
	S13S	1, (pS13S)	~89	pS13-2	1.06×10^–4^	IncX1	*bla*_CTX–M–55_

*The transconjugant S13D was selected on MacConkey agar plates supplemented with rifampin (400 mg/L) and doxycycline (16 mg/L), S13F was selected on MacConkey agar plates supplemented with rifampin (400 mg/L) and florfenicol (16 mg/L), S13S was selected on MacConkey agar plates supplemented with rifampin (400 mg/L) and ceftiofur (16 mg/L).*

### Sequence Analysis of Plasmids in *S. enterica* Strain S13

**FIGURE 1:**
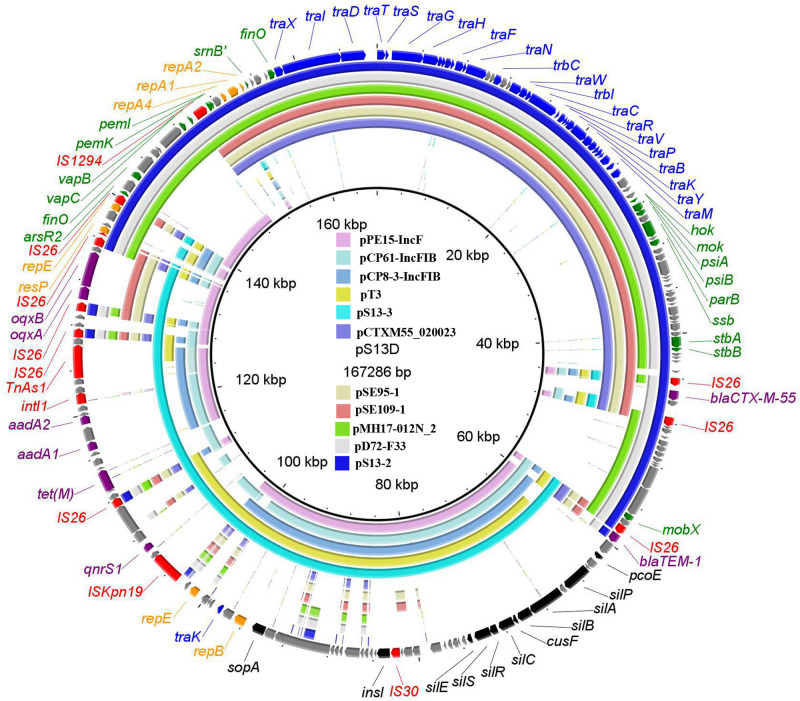
The whole-plasmid sequence of pS13D and comparisons of pS13-2 and pS13-3, with similar plasmids. The outer ring comprises the CDSs of pS13D. The plasmids in this study included pS13D (MT657397), pS13-2 (CP047092), pS13-3 (CP047093), pCTXM55_020023 (CP025949), pSE95-1 (CP050717), pSE109-1 (CP050710), pMH17-012N_2 (AP018568), pD72-F33 (CP035314), pPE15-IncF (CP041629), pCP61-IncFIB (CP053729), pCP8-3-IncFIB (CP053738), and pT3 (MK656937). Key features of pS13D are highlighted in different colors. Replicon genes are in highlighted in orange, transfer-associated genes in blue, resistance genes in purple, mobile elements in red, virulence related genes in black, and hypothetical proteins in gray.

WGS showed that strain S13 has a 4, 744, 425-bp chromosome (G+C content, 50.96%) and four plasmids, pS13-1 (96, 320 bp), pS13-2 (89,179 bp), pS13-3 (77, 279 bp), and pS13-4 (30, 069 bp). The P7 phage-like plasmid pS13-1 carried the *mcr-1* resistance gene. BLASTn comparisons showed that pS13-1 was highly similar to the *mcr-1*-carrying plasmid, as determined by strict structural analysis (Supplementary Figure 2). The pS13-1 plasmid was not self-transmissible and failed to bind to an auxiliary plasmid, although pS13-1-like plasmids are reportedly transferrable *via* insertion into a conjugative plasmid (He et al., 2019). The plasmid pS13-2 harboring the *bla*_CTX–M–55_ gene was a type of IncN1-F33:A–:B– plasmid and possessed the typical backbone of F33:A–:B– plasmids; carried resistance genes and mobile elements in the MDR region, including *bla*_CTX–M–55_, *Δbla*_*TEM–1*_, IS*26*, and IS*1294*; and exhibited high homology to other plasmids in *E. coli*, *Citrobacter freundii*, and *S*. *enterica* (Figure 1).

The *tet*(M)-bearing plasmid pS13-3 was a novel IncFIA-FIB plasmid possessing the typical structure of IncFIB-type plasmids but lost the *tra* gene in the transfer region. Online BLASTn analysis revealed that pS13-3 exhibited homology to the *E. coli* plasmids pPE15-IncF (CP041629), pCP61-IncFIB (CP053729), pCP8-3-IncFIB (CP053738), and pT3 (MK656937) shared 99% identity at 72–84% coverage. As compared with the plasmids mentioned above, the most visible difference of strain S13 was the MDR, which comprised three accessory modules, including the novel *tet*(M)-bearing transposon Tn*6942*, the *oqxAB* resistance module *bleO*-*NimC*-IS*26*-*oqxAB*-IS*26*-*bla*_*TEM–1*_, and the segment IS*Kpn19*-*TinR*-*qnrS1* (Figure 1).

**FIGURE 2:**
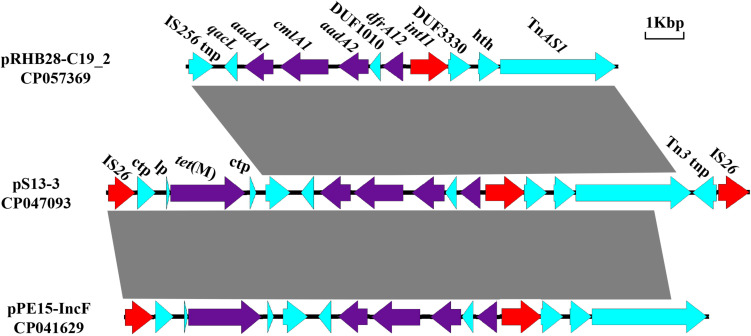
Comparisons of the genetic environment of Tn*6942* harboring *tet*(M) in CP047093, with two other sequences retrieved from the GenBank database. Similar regions are indicated by dotted lines (lp, gene encoding *tet*(M) leader peptide; tnp, transposage; hth, helix-turn-helix domain-containing protein).

The novel composite transposon Tn*6942* (16, 493 kb), harboring the *tet*(M) resistance gene, and an integron carrying *IntI1*, *aadA1*, *cmlA1*, *aadA2*, and *dfrA12* was flanked by two IS*26* elements. Tn*6942* was highly similar to that of a fragment in pPE15-IncF recovered from *E. coli* strain pPE15 in Henan, China. The main difference is that the downstream Tn*AS1* in Tn*6942* was replaced with *bla*_*TEM–1*_ in plasmid pPE15-IncF. Meanwhile, plasmid pRHB28-C19_2 (CP057369) had a similar structure but lacked two parts of segments at both ends, including the *tet*(M) resistance module [IS*26*-conjugal transfer protein-LP-*tet*(M)] and a Tn*3* family transposase (Figure 2). Reverse PCR analysis was performed to assess the existence of a circular intermediate of Tn*6942* (Supplementary Table 2). There is a circular intermediate IS*26*-bracked composite transposon carrying *tet*(M), excising from the S13 strain, revealing that the Tn*6942* could stimulate the dissemination of *tet*(M) gene.

**FIGURE 3:**
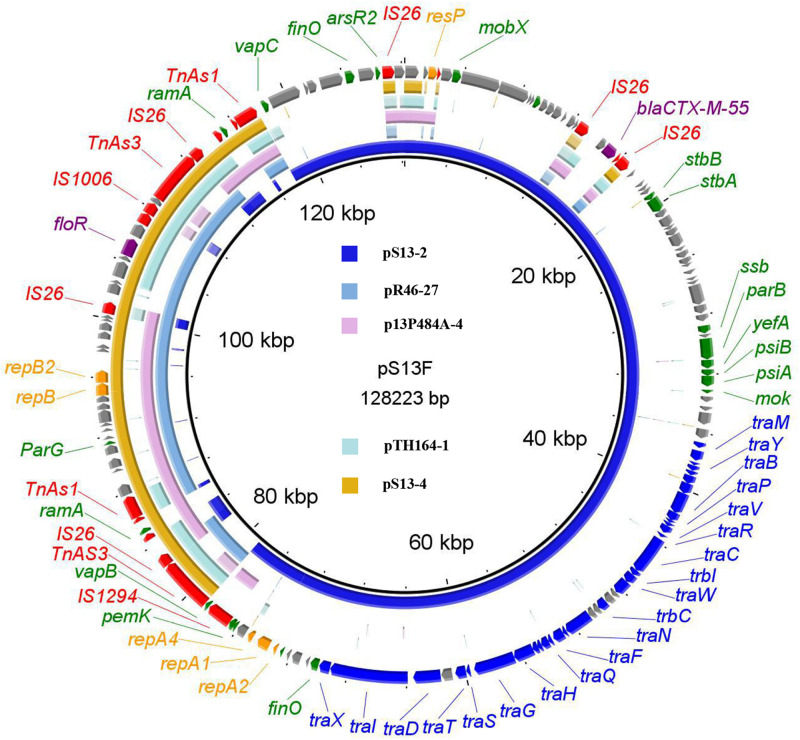
The whole-plasmid sequence of pS13F, comparison of pS13-2 and pS13-4 with similar plasmids. The outer ring comprises the CDSs of pS13F. The plasmids in this study included pS13F (MT742153), pS13-2 (CP047092), pS13-4 (CP047094), pR46-27 (CP035774), p13P484A-4 (CP019284), and pTH164-1 (CP035212). Key features of pS13F are highlighted in the same color as the pS13D.

The *floR*-bearing IncX1 plasmid pS13-4 comprised 42 open reading frames. BLASTn analysis showed that plasmid pS13-4 was most similar to the IncX1 plasmid pR46-27 (CP035774), with 99% identity at 91% coverage (Figure 3). The plasmids pS13-3 and pS13-4 both lost the *tra* gene region responsible for plasmid conjugation, which may be the reason why these two plasmids were not self-transmissible. However, once inserted into the successfully diffused conjugative plasmid, all resistance genes carried by the plasmids achieved efficient movement. Two cases in point are the formation of conjugative fusion plasmids.

### Proposed Formation Mechanism of Two Fusion Plasmids

**FIGURE 4:**
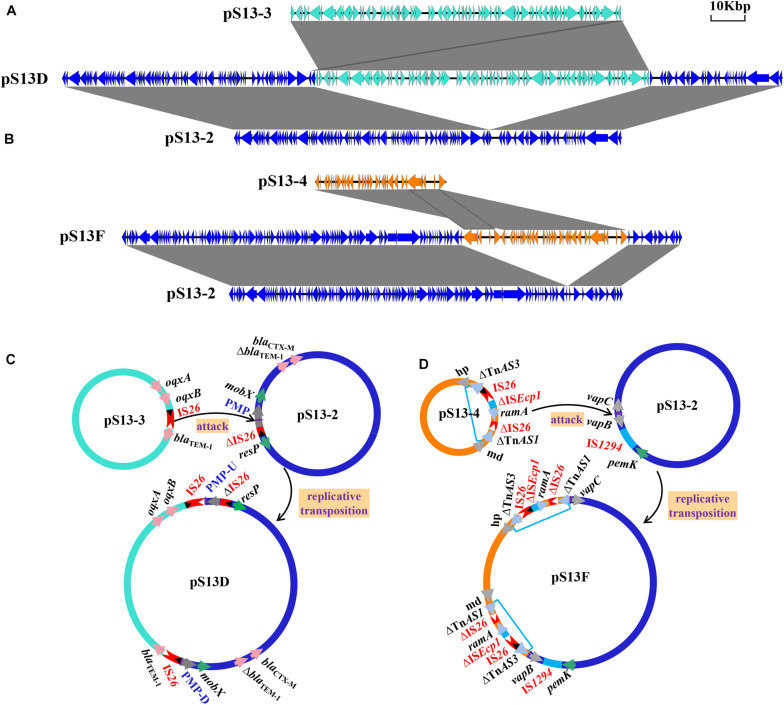
The mechanism of plasmid fusion, including pS13D and pS13F. The shaded area indicates 100% identity. Colored arrows indicate open reading frames, with pS13-2 in blue, pS13-3 in turquoise, and pS13-4 in orange. **(A)**. Linear sequence comparison of pS13-2, pS13-3, and the fusion plasmid pS13D. **(B)**. Linear sequence comparison of pS13-2, pS13-4, and the fusion plasmid pS13F. **(C)**. A model for the formation of pS13D mediated by IS*26*. **(D)** A model for the formation of pS13F mediated by Tn*AS3*-IS*26*-*Δ*IS*Ecp1*-*ramA*-*Δ*IS*26*-*Δ*Tn*AS1*. Plasmid names are shown in black. ISs are shown as rectangles (different colors represent different ISs), with triangles representing the terminal inverted repeats (TIRs). Left TIRs, open triangle; right TIRs, closed triangle; pink arrows, resistance genes; gray arrows, proteins. Purple arrows indicate different flanking 8-bp sequences and 5-bp sequences.

The plasmid pS13D harboring the *tet*(M) resistance gene was found to be 167,286 kb in size with a G+C content of 51% and contained 173 conserved domains. Further analysis indicated that pS13D was a fusion plasmid with chimeric characteristics consisting of pS13-2, pS13-3, an additional IS*26* copy, and an 8-bp sequence target site duplication (TSD) (TTCAAGAT) (Figure 4A). The sequences across the cointegrate junctions were confirmed with the primers Fusion-168-1/2-F/R to link the pS13-3 backbone to the pS13-2 backbone, and the sequences of the PCR amplicons were consistent with that of WGS (Supplementary Table 1). Based on the above sequence analysis, we propose the fusion and resolution model of pS13D shown in Figure 4C. In this model, IS*26* between *bla*_*TEM–1*_ and *oqxB* in pS13-3 captured the conjugative plasmid pS13-2 through attack of a putative mobilization protein (PMP) in the *mob*A/*mob*L family, and intermolecular transposition occurred *via* a replicative transposition mechanism, leading to an 8-bp TSD and an additional copy of IS*26* flanking the pS13-3 molecule located upstream of PMP-U. Genetic rearrangement continued as the novel IS*26* attacked the TSD (TTCAAGAT) downstream of PMP-D, which subsequently resulted in two TSDs surrounding the insertion fragment and eventually the formation of the hybrid resistance plasmid pS13D.

IS*26*, which belongs to the IS*6* family of mobile elements, plays a pivotal role in the spread, clustering, and recombination of resistance genes (He et al., 2015; Liu Z. et al., 2020). Examples of the phenomenon of plasmid fusion mediated by IS*26* include fusion of IncF33:A–:B– and phage-like plasmids, IncFII and the IncX3 plasmid, IncHI2 and IncFIB plasmids (Chen et al., 2017; Harme and Hall, 2017; Liu Z. et al., 2020). Significantly, the recombination mechanisms of the plasmids described above were all related to the IS*26* element located on conjugative plasmids attacking the non-conjugative plasmids. In this study, the non-conjugative IncFI:A–:B–type plasmid captured a IncN1-F33:A–:B– type conjugative helper plasmid, which was mediated by the IS*26* element, similar to the fusion of the IncX1 and IncI1 plasmids (Chen et al., 2019).

The hybrid plasmid pS13F harboring the *floR* gene in the transconjugant S13F was shown to be 128,223 bp in size and belonged to the IncN1-F33:A–:B–/IncX1 plasmid. Sequence alignment revealed that pS13F was obtained through the fusion of pS13-2 (1–78,755 nt; 117,800–128,223 nt) and pS13-4 (78,756–108,824 nt) (Figure 4B). Interestingly, pS13F carried an extra 8970-bp sequence (108,825–117,794 nt) containing a fragment of *Δ*Tn*AS3*-IS*26*-*Δ*IS*Ecp1*-*ramA*-*Δ*IS*26*-*Δ*Tn*AS1* and an additional 5-bp sequence (TTATA). Based on sequence comparison, the plasmid recombination was mediated by a segment of *Δ*Tn*AS3*-IS*26*-*Δ*IS*Ecp1*-*ramA*-*Δ*IS*26*-*Δ*Tn*AS1*, designated Tn*6952*, which was located on plasmid pS13-4 (Figure 3 and Supplementary Figure 1). As shown in the model, the 8970-bp segment Tn*6952* adjacent to the malate dehydrogenase coding region of pS13-4 attacked the target site (TTATA) of pS13-2 prior to replicative cointegrate formation. Linearized pS13-2 was incorporated into pS13-4 creating the cointegrate pS13F, giving the appearance of a 5-bp TSD (TTATA) and acquired an additional copy of Tn*6952* located downstream of the incoming pS13-2 molecule. Subsequently, the TSDs became two direct repeats around the insertion fragment (Figure 4D). To confirm the genetic structures, two primers (Fusion-128-1/2-F/R) were designed, targeting the corresponding fusion regions. Notably, the sequences of the PCR amplicons were consistent with those obtained by WGS, which certified the existence of fusion regions (Supplementary Table 1).

**FIGURE 5:**
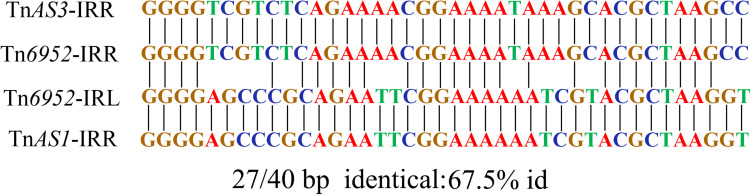
Alignment of the IRR and IRL in Tn*6952* with the IRs identified on the Tn*AS3* and Tn*AS1*.

The Tn*6952* contained an intact IS*26* sequence, two truncated insertion sequences (*Δ*IS*Ecp1* and *Δ*IS*26*), the AraC/XylS family gene *ramA*, and two Tn*3*-like element family transposases at both ends. Comparative analysis demonstrated that the segment ΔTn*AS3*-IS*26* existed in *Klebsiella pneumoniae* plasmid pR46-27 and *E. coli* plasmid unnamed1 (CP037904). In addition, the fragment ΔIS*Ecp1*-*ramA*-ΔIS*26*-ΔTn*AS1* was observed in *E. coli* plasmids p13P484A-4 (CP019284) and pEC129_3 (CP038456), and noteworthy, no direct repeat was identified at each extremity of the Tn*6952*, suggesting that Tn*6952* acquired by pS13-4 may have occurred by recombination. To date, there has no report of plasmid fusion mediated by such complex structures, as only the segment of Tn*3* carrying mutations in the repressor gene *tnp*R was able to mediate cointegration of the plasmids, as reported by Ohtsubo et al., who proposed that the repressor could destabilize the cointegrated plasmids through the action of an internal resolution site, while the transposase *tnpA* and inverted repeat right (IRR) may have taken part in mediating the formation of cointegrated plasmids containing two direct repeats of Tn*3* (Ohtsubo et al., 1981). Further analysis showed that both boundaries [IRR and inverted repeat left (IRL)] of Tn*6952* shared 100% identity with the IRR of two Tn*3*-like elements, Tn*AS3* and Tn*AS1*, respectively (Figure 5). While in transposon Tn*6952*, the IRR shared 67.5% nucleotide identity with the IRL. The Tn*AS3* (Kieffer et al., 2019) and *Δ*Tn*AS1* (Liu et al., 2017) transposases could mobilize the resistance genes *mcr-3* and *mcr-5* by IRs, respectively. So, we speculate that the two IRs and transposase of Tn*AS3* and ΔTn*AS1* may be involved in the molecular transposition of plasmid pS13-4 through forming an efficient mobilization unit Tn*6952*. The *ramA* gene, which controls expression of the MDR efflux pump genes and influences the virulence of *S. enterica* could be the facilitator that regulates the stability of Tn*6952* (Bailey et al., 2010; Ricci et al., 2014). Therefore, Tn*6952* carrying three insertion sequences may accelerate the dissemination of resistance genes and plasmids by cointegration.

### The Biological Features of Two Fusion Plasmids

Stability assays showed that two fusion plasmids, pS13D and pS13F, were appeared to be stable (stability=100%) in *E. coli* for at least 10 days of passage in an antibiotic-free environment. For the reason that cointegrate pS13D and pS13F could be transferred from the transconjugant S13D and S13F to the recipient C600, the experiments of fitness cost of pS13D and pS13F could not performed accurately. However, to some extent, two fusion plasmids did not exhibit a fitness cost to *E. coli C600* (Supplementary Experiments). In addition, pS13D and pS13F were transferred to the *E. coli* strain J53 at high conjugation frequencies of 8.58×10^–3^ and 9.2×10^–4^, respectively, indicating that these two plasmids had relatively high self-transmission rates and stability, and contributed to the dissemination of the resistance genes *tet*(M), *oqxAB*, and *floR via* co-selection, thereby posing a potentially threat to clinical treatment.

## Conclusion

A novel IncN1-F33:A–:B– conjugative helper plasmid was identified that could passively fuse with non-conjugative tetracycline and florfenicol resistance-encoding plasmids *via* two different mechanisms, thereby promoting the conversion into conjugative plasmids transmissible among different species of *Enterobacteriaceae*. A better understanding of the molecular and triggering mechanisms that contribute to the formation and evolution of cointegration of MDR plasmids is needed to build a foundation to curb further spread of resistance elements among bacterial pathogens *via* this type of plasmid. New intervention measures are urgently needed to curb formation and dissemination of such elements among *Salmonella* species and other *Enterobacteriaceae*.

## Data Availability Statement

The datasets presented in this study can be found in online repositories. The names of the repository/repositories and accession number(s) can be found in the article/Supplementary Material.

## Author Contributions

G-ZH, J-HL, and D-DH conceived and designed the experiments. Y-YL and M-KZ performed the experiments. Y-YL analyzed the data. Y-SP, J-HL, HW, and LY contributed reagents, materials, and analysis tools. Y-YL, D-DH, Y-SP, and G-ZH wrote the manuscript. All authors contributed to the article and approved the submitted version.

## Conflict of Interest

The authors declare that the research was conducted in the absence of any commercial or financial relationships that could be construed as a potential conflict of interest.

## Footnotes

1 https://mic.eucast.org/Eucast2/

## Supplementary Material

Supplementary Figure 1 **(a)** S1-PFGE of *S. enterica* strain S13 and its three transconjugants S13D, S13F, S13S. **(b)**. Southern hybridization of *S. enterica* strain S13 and the *tet*(M)-bearing transconjugant S13D with the *tet*(M) gene as the probe. Marker, *Salmonella Braenderup* H9812.

Supplementary Figure 2 Structural comparisons of pS13-4 and the similar plasmids. The outer ring represents the CDSs of the reference sequence pS13-4. The similar plasmids including the *E. coli* plasmids pD72-mcr1 (CP035313), pZR78 (MF455226), and pGD27-62 (MN232192); *S. enterica* plasmid pATCC10729_02 (CP019188); and *Klebsiella pneumoniae* plasmids pMCR_SCKP-LL83 (MF510496) and p160070 -MCR (MG288678).

Supplementary Table 1 Primers used for PCR and DNA sequencing in this study.

Supplementary Table 2 The MICs of S13 and its transconjugants in this study.
