# Supplementary material for: The Formation of Two Hybrid Plasmids Mediated by IS26 and Tn6952 in Salmonella enterica Serotype Enteritidis
Source: Front Microbiol. 2021 May 28;12:676574. doi: 10.3389/fmicb.2021.676574 (PMC8193513; doi:10.3389/fmicb.2021.676574)
Supplement: Supplementary Figure 1 — (a) S1-PFGE of S. enterica strain S13 and its three transconjugants S13D, S13F, S13S. (b). Southern hybridization of S. enterica strain S13 and the tet(M)-bearing transconjugant S13D with the tet(M) gene as the probe. Marker, Salmonella Braenderup H9812. [file Data_Sheet_1.ZIP › Supplementary files/Supplementary Experiments.docx]

**Supplementary Experiments**

**Methods**

The fitness cost of plasmids pS13D and pS13F were determined by two competition experiments, *E. coli* transconjugant S13D and C600, *E. coli* transconjugant S13F and C600, as previously described (San et al., 2009). Brief speaking, S13D (S13F) and *E. coli* C600 was grown for 16 h at 37℃ in 20 mL LB broth. Then 2×10^6^ CFU S13D (S13F) was mixed with *E. coli* C600 equally in 20 mL antibiotic-free LB broth and incubated at 37℃ with continuous shaking (120 rpm). A total of 4×10^6^ CFU was transferred to 20 mL fresh LB broth every 24 h. Samples were taken every hour during the first 12 h, at 24 h, and then every 24 h for 5 days. The numbers of CFU for every samples in LB agar plates with and without doxicycline (florfenicol) were determined.

**Results**

The resauls were shown in Figures as follows, equal numbers of bacteria with and without plasmid pS13D were mixed and subcultured every 24 h for 5 days, and the proportion of bacteria carrying this plasmid was monitored every 4 hours for the first 12 h. For this period, the ratio was almost 1:1 at every point, whereas the S13D outnumbered the *E. coli* C600 after co-culture for 1 day. After that, the numbers of *E. coli* C600 in mix-culture continuously decreased. At 5 day, the number of C600 was less than 1% of its culture (Figure. a). Like S13D, S13F outnumbered the *E. coli* C600 after 1 day, while until the 6 day, the number of C600 was decreased to less than 1% of its culture. The transconjugants S13D and S13F showed competitive advantage relative to *E. coli* C600 in the entire experiment (Figure. b).

In conclusion, there is no evidence showed that the two transconjugants harbouring fusion plasmids exhibited fitness cost to *E. coli* C600. These results do not rule out that the decline of *E. coli* C600 might be due to the conjugation of pS13D and pS13F from the transconjugant S13D (S13F) to the recipient C600. Therefore, it is impossible to draw a conclusion whether the two fusion plasmids exhibited fitness cost to *E. coli* C600, because of the two fusion plasmids could transfer. while it is indicated to some extent that two fusion plasmids did not cause fitness cost to *E. coli* C600, because of the two fusion plasmids could transfer.


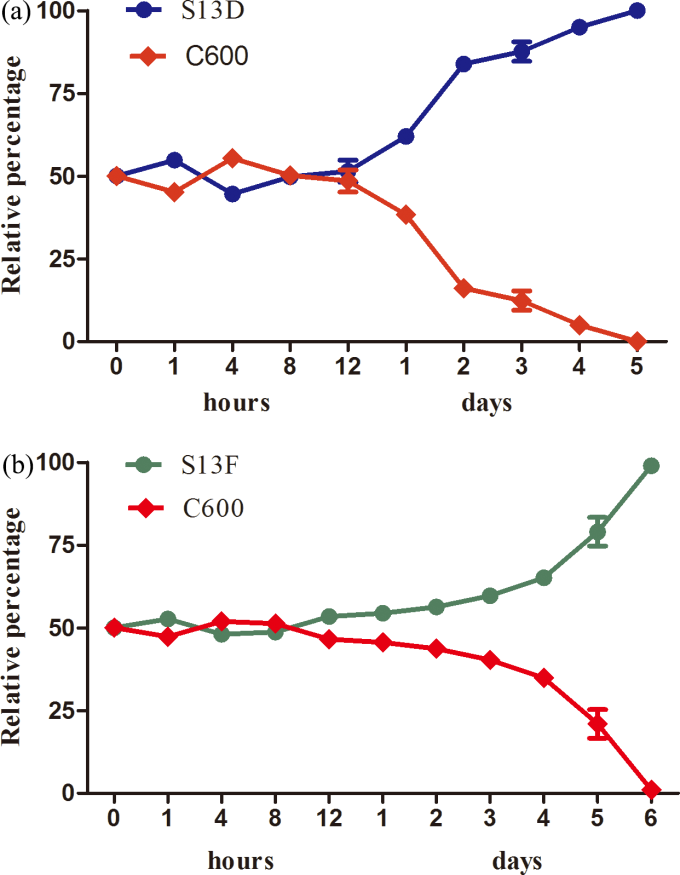


**Figure. The fitness cost of S13D and S13F.** (a). Competition profiles between S13D and C600, the initial ratio was equal. (b). Competition profiles between S13F and C600, the initial ratio was equal. The relative percentage of each strain at different time points is shown. The competition curves were constructed with the mean of three independent experiments that the standard deviation represented by error bars.

**References**

San Millan, A., Escudero, J. A., Gutierrez, B., Hidalgo, L., Garcia, N., Llagostera, M., et al. (2009). Multiresistance in pasteurella multocida is mediated by coexistence of small plasmids. *Antimicrobial Agents and Chemotherapy*. 53, 3399-3404.

Wu, R., Yi, LX., Yu, LF., Wang, J., Liu, Y., Chen, X., et al. (2018). Fitness Advantage of *mcr-1*-Bearing IncI2 and IncX4 Plasmids *in Vitro*. *Front Microbiol*. Feb 27; 9: 331.
