# Supplementary material for: The Formation of Two Hybrid Plasmids Mediated by IS26 and Tn6952 in Salmonella enterica Serotype Enteritidis
Source: Front Microbiol. 2021 May 28;12:676574. doi: 10.3389/fmicb.2021.676574 (PMC8193513; doi:10.3389/fmicb.2021.676574)
Supplement: Supplementary Figure 1 — (a) S1-PFGE of S. enterica strain S13 and its three transconjugants S13D, S13F, S13S. (b). Southern hybridization of S. enterica strain S13 and the tet(M)-bearing transconjugant S13D with the tet(M) gene as the probe. Marker, Salmonella Braenderup H9812. [file Data_Sheet_1.ZIP › Supplementary files/Tables.docx]

Table S1. Primers used for PCR and DNA sequencing in this study

| Gene | Primer name | sequences(5’ to 3’) | Size(bp) | Reference |
| --- | --- | --- | --- | --- |
| Fusion-167-1 | 167-1-F | CTGAAACTCCTCAGTCAGCGT | 947 | this study |
|  | 167-1-R | CGTCTGACGTTGAACACCGAC |  |  |
| Fusion-167-2 | 167-2-F | AACCATCTGCAAAGCTGTCG | 1198 | this study |
|  | 167-2-R | CATGGATTTAAATCCCAGCGTG |  |  |
| Fusion-128-1 | 128-1-F | ACCGCCTTGGGGAAATCAG | 701 | this study |
|  | 128-1-R | TAGCTTCGCAGGTTCATCATCC |  |  |
| Fusion-128-2 | 128-2-F | CAGTGGCGACCTTAACACCATAT | 1105 | this study |
|  | 128-2-R | GGGCTTCACCGATCACGTCTTC |  |  |
| *bla*_CTX-M_ | CTX-M-F | GGGCTGAGATGGTGACAAAGAG | 861 | 1 |
|  | CTX-M-R | CGTGCGAGTTCGATTTATTCAAC |  |  |
| *floR* | floR-F | ATGCAGAAGTAGAACGCG | 868 | 2 |
|  | floR-R | ATGCAGAAGTAGAACGCG |  |  |
| *tet*(M) | *tet*(M)-F | GTGGACAAAGGTACAACGAG | 406 | 3 |
|  | *tet*(M)-R | CGGTAAAGTTCGTCACACAC |  |  |
| *mcr-1* | mcr-1-F | ATGATGCAGCATACTTCTGTG | 1626 | 4 |
|  | mcr-1-R | TCAGCGGATGAATGCGGTG |  |  |
| ReverseTn | RTn-F | CTCTGTAATGAGTTGGTCTA | 1049 | this study |
|  | RTn-R | CACGAGTTAGCGGAACCATT |  |  |

[1] Wu, H., Liu, B.G., Liu, J.H., Pan, Y.S., Yuan, L., Hu, G.Z., 2011. Phenotypic and molecular characterization of CTX-M-14 extended-spectrum β-lactamase and plasmid-mediated ACT-like AmpC β-lactamase produced by *Klebsiella pneumoniae* isolates from chickens in Henan Province, China [J]. Genetics & Molecular Research. 11(3), 3357-3364.

[2] Li, B., Zhang, Y., Wei, J., Shao, D.H., Liu, K., Shi, Y.Y., Qiu,Y.F., Ma, Z.Y., 2015. Characterization of a novel small plasmid carrying the florfenicol resistance gene *floR* in *Haemophilus parasuis* [J]. 70(11), 3159-61.

[3] Sun Y.W., Liu Y.Y., Wu H et al. IS*26*-Flanked Composite Transposon Tn*6539* Carrying the *tet*(M) Gene in IncHI2-Type Conjugative Plasmids From *Escherichia coli* Isolated From Ducks in China [J]. *Frontiers in microbiology* 2018; 9: 3168.

[4] Li, X.S., Liu, B.G., Dong, P., Li, F.L., Yuan, L., Hu, G.Z., 2018. The prevalence of mcr-1 and resistance characteristics of *Escherichia coli* isolates from diseased and healthy pigs [J]. Diagnostic Microbiology and Infectious Disease. 91(1), 63-65.

Table S2. The MICs of S13 and its transconjugants in this study

| Isolates | AMC | CF | CEQ | GM | AMK | TET | OXY | DOX | FFC | COL | ST | ENR |
| --- | --- | --- | --- | --- | --- | --- | --- | --- | --- | --- | --- | --- |
| S13 | >512 | >512 | 256 | <0.5 | <0.5 | 256 | 256 | 32 | 256 | 4 | 256 | <0.5 |
| S13D | >512 | 256 | 128 | <0.5 | <0.5 | 16 | 16 | 16 | 8 | <0.5 | 256 | <0.5 |
| S13F | >512 | 256 | 128 | <0.5 | <0.5 | 16 | 8 | 8 | >512 | <0.5 | 256 | <0.5 |
| S13S | >512 | >512 | 128 | <0.5 | <0.5 | 1 | <0.5 | <0.5 | 1 | <0.5 | 256 | <0.5 |
| C600 | 1 | <0.5 | <0.5 | <0.5 | <0.5 | 1 | 0.5 | 0.5 | 1 | <0.5 | 1 | <0.5 |

AMC, Amoxicillin; CF ceftiofur; CEQ, Cefquinome; GM, Gentamicin; AMK, amikacin; TET, Tetracycline; OXY, oxytetracycline; DOX, doxycycline; FFC, florfenicol; COL, Colistin; ST, trimethoprim/sulfamethoxazole; ENR, Enrofloxacin.
