# Supplementary figures and images for: The Formation of Two Hybrid Plasmids Mediated by IS26 and Tn6952 in Salmonella enterica Serotype Enteritidis
Source: Front Microbiol. 2021 May 28;12:676574. doi: 10.3389/fmicb.2021.676574 (PMC8193513; doi:10.3389/fmicb.2021.676574)

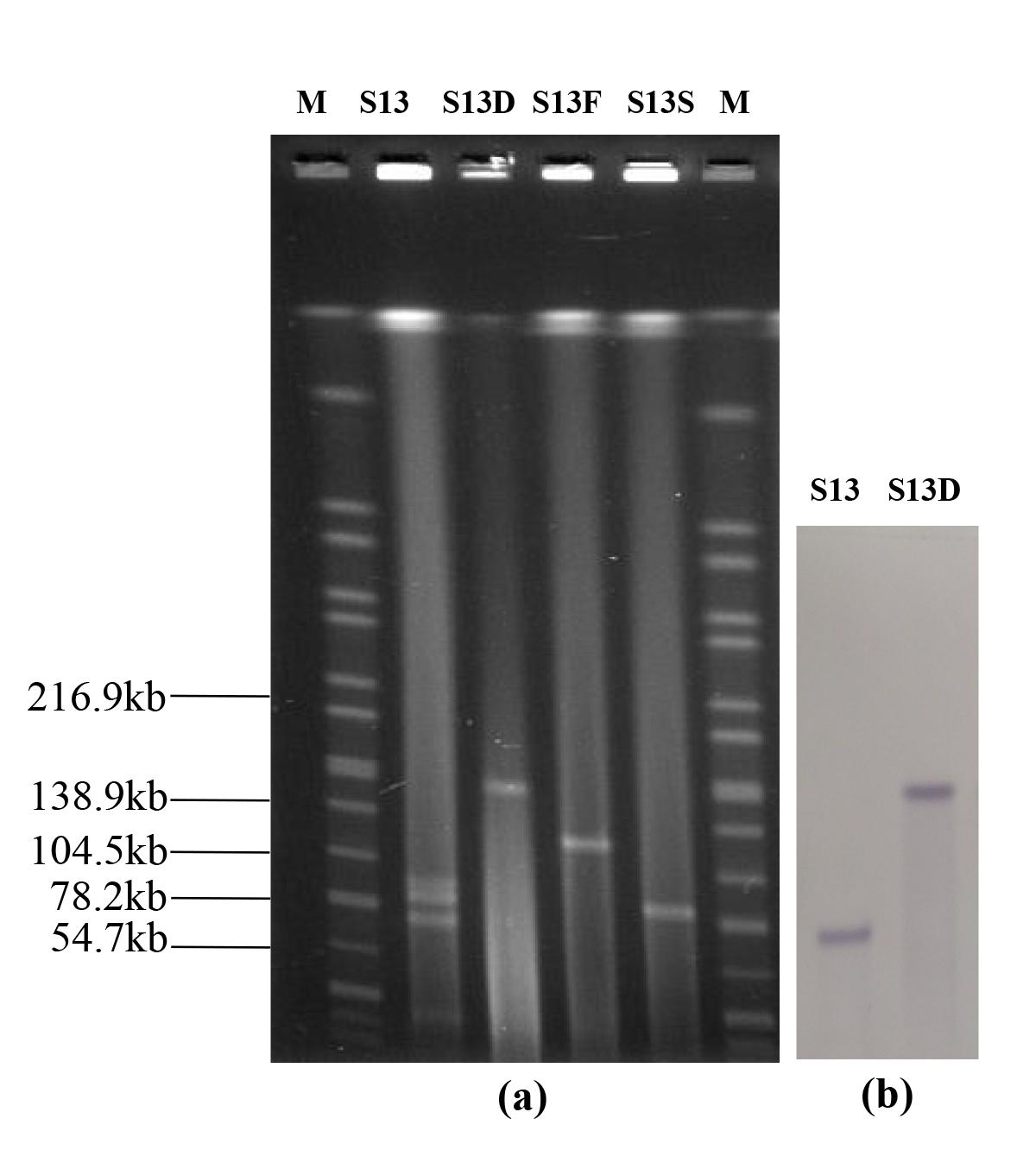

Supplement: Supplementary Figure 1 — (a) S1-PFGE of S. enterica strain S13 and its three transconjugants S13D, S13F, S13S. (b). Southern hybridization of S. enterica strain S13 and the tet(M)-bearing transconjugant S13D with the tet(M) gene as the probe. Marker, Salmonella Braenderup H9812. [file Data_Sheet_1.ZIP › Supplementary files/Figure.S1.tif]

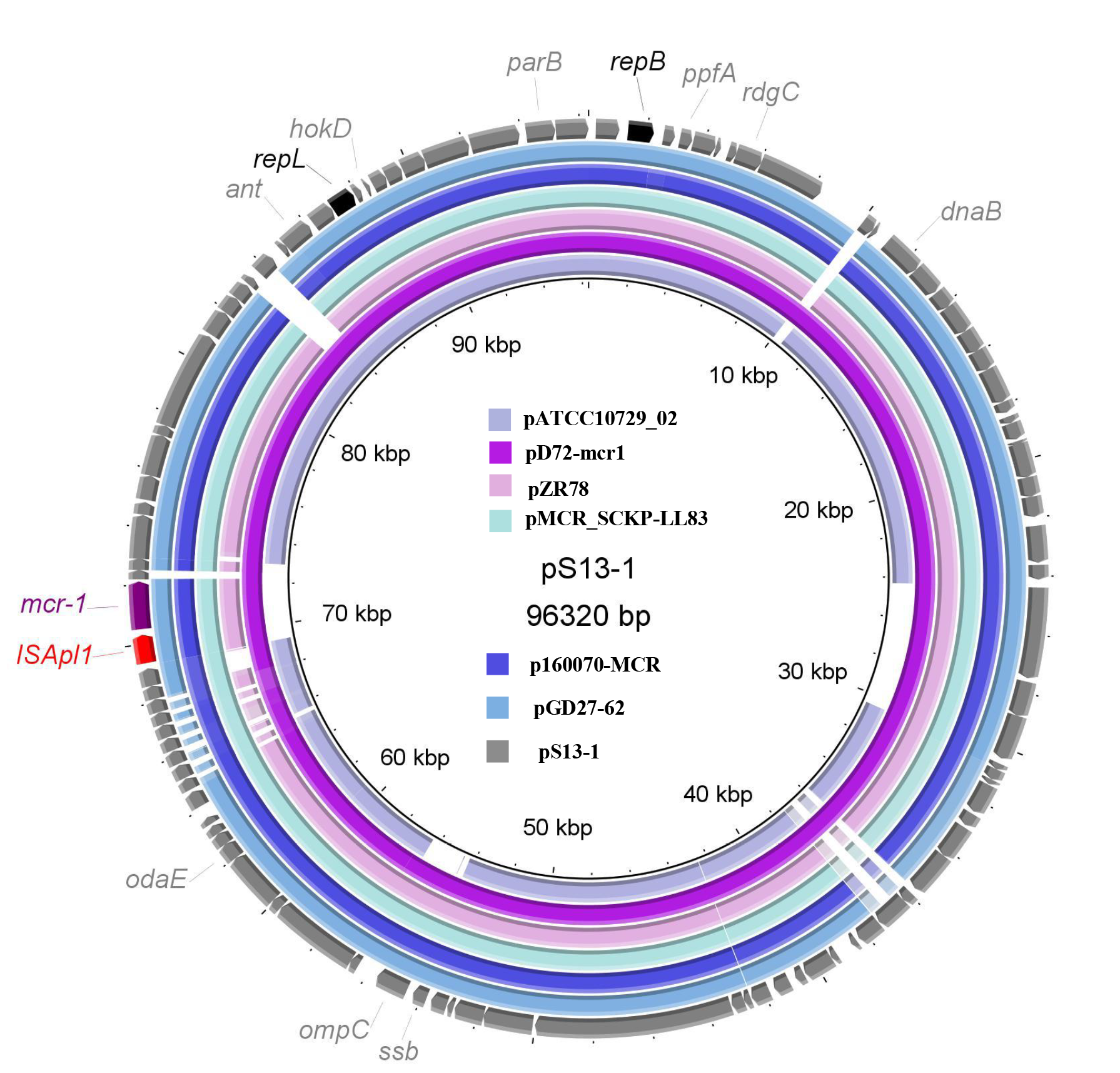

Supplement: Supplementary Figure 1 — (a) S1-PFGE of S. enterica strain S13 and its three transconjugants S13D, S13F, S13S. (b). Southern hybridization of S. enterica strain S13 and the tet(M)-bearing transconjugant S13D with the tet(M) gene as the probe. Marker, Salmonella Braenderup H9812. [file Data_Sheet_1.ZIP › Supplementary files/Figure.S2.tif]
